# Fattening performance and carcass traits of Baladi and Shami-Baladi kids

**DOI:** 10.14202/vetworld.2020.21-25

**Published:** 2020-01-03

**Authors:** Mohammad D. Obeidat, Belal S. Obeidat, Basheer Nusairat, Rolan Al-Shareef

**Affiliations:** 1 Department of Animal Pro, Jordan University of Science and Technology, Irbid 22110, Jordan; 2 Department of Nutrition and Food Technology, Jordan University of Science and Technology, Irbid 22110, Jordan

**Keywords:** crossbred, goat, Shami

## Abstract

**Background and Aim:**

Goat is considered very important ruminant animals that are raised in Jordan; however, the production of the local breeds is low to moderate, and thus, some genetic improvement programs could have a positive effect on the performance of the local breeds. The objective of this study was to investigate the effect of crossing Baladi (BB) goat with Shami.

**Materials and Methods:**

A total of 30 newly weaned goat kids (15 BB and 15Shami-Baladi [SB] kids) were evaluated for growth performance and carcass traits. The trial lasted for 77 days (7 days for adaptation and 70 days for data collection). Feed intake was measured on a daily basis; body weight of kids was measured at the beginning of the study and biweekly thereafter. At the end of the trial, kids were slaughtered to examine carcass traits.

**Results:**

Dry matter intake, initial weight, final weight, and average daily gain (ADG) were not affected by kid’s breed type. In addition, breed type of kids showed no significant effect on feed efficiency (feed:gain). Hot and cold carcass weight and dressing percentage were also not affected by kid’s breed type. Kid breed type had no significant effect on shoulder, rack, and legs percentages. However, SB kids had a higher loin cut percentage compared to BB kids. In addition, *Musculus longissimus* depth was higher for SB kids.

**Conclusion:**

Results of this study indicate that crossing SB goat breeds did not have a significant effect on either growth performance or carcass traits. Further studies using reciprocal cross may reveal different results.

## Introduction

Goats can adapt and be productive in different types of agro-climatic zone and they also have the potential to satisfy the protein requirement of a growing human population [1]. Moreover, due to some health considerations and consumer preferences, the demand for goat meat has increased during the past several years. Goat meat had lower levels of saturated fatty acids and cholesterol, and higher levels of unsaturated fatty acids compared to lamb meat [2]. In addition, Sen *et al*. [3] reported that goat yielded leaner carcasses when compared to sheep. However, meat production of local goat breeds is inadequate to meet the high demand. The average mature weight of local goat breeds ranges from 32 kg in Dhaiwi to 40 and 46 kg in Desert and Mountain goat, respectively [4]. As a result, genetic improvement programs were initiated to improve the productivity of local goats. Growth parameters such as body weight (BW) and average daily gain (ADG) are important traits for measuring the effectiveness of selection programs. Selection is mainly based on BW between 6 and 12 months of age, with increasing BW considered an important goal [5]. However, crossbreeding is still considered the fastest way to improve the productivity of local breeds.

Some exotic breeds with noticeable meat and milk production have been imported to Jordan and raised as pure breeds or crossed with local goats. During the past decade, the most famous exotic breed that has been widely used in Jordan is Shami goat. Damascus (Shami) goat originated from Syria, Cyprus, Turkey, and other Mediterranean countries. The breed is considered to be the most predominant and important dual-purpose breed in the Middle East countries under semi-intensive or intensive production systems [6]. The significance of the current study is that it evaluates the performance of the Damascus crossbred kids which have been widely raised by producers.

The present study aimed to compare the growth and carcass traits of Baladi (BB) and Shami-Baladi (SB) crossbred kids.

## Materials and Methods

### Ethical approval

Procedures used in the current study were approved by Jordan University of Science and Technology (JUST) Institutional Animal Care and Use Committee, approval number 15/3/3/490.

### Animals

The study was conducted in the Northern part of Jordan at the Agricultural Research and Training Unit at JUST. A total of 30 single born goat male kids were weaned at 75 days of age and divided into two groups according to their breed type (15 BB and 15 SB kids). Data collection started after 7-day adaptation [7] of kids to the diet and experimental area. All kids were fed the same diet and water *ad libitum* for 70 days. At the beginning of the trial, fasting BW of kids was taken in the morning. Live BW through the trial period was recorded on biweekly intervals, and feed intake was measured on a daily basis.

### Slaughter procedure and carcass traits

At the end of the feeding period of the trial, kids from each genetic group were slaughtered after fasting 12 h and final live BW was recorded. Kids were slaughtered by cutting the arteries and the jugular vein in the neck region and left to bleed for a few minutes. All non-carcass components such as heart, lungs, kidneys, spleen, and gastrointestinal tract were also weighed. Hot carcass weight was recorded after washing the carcass with freshwater. Carcasses were kept in the chiller for 24 h at 4°C and then cold carcass weights were recorded. Each carcass was cut into four parts (leg, shoulder, loin, and rack) following the procedure described by Abdullah *et al*. [8]. Each cut was weighed and then tissue depth (GR), rib fat depth (J), leg fat depth (L3), shoulder fat depth (S2), area of *Musculus* *longissimus* (LMA), eye muscle width (A), eye muscle depth (B), and fat depth (C) were recorded. The right leg cut was then sealed into plastic bags and frozen at −20°C until dissected.

Before dissection, legs were thawed in cooler at 4°C in plastic bags. Leg was dissected and subcutaneous fat, intermuscular fat, muscle, bone, and scrap were separated and weighed. Meat quality parameters (pH, color [CIE l, a, b coordinates], cooking loss [CL], water holding capacity [WHC], and shear force [SF] values) were measured following the procedure described by Obeidat *et al*. [7].

### Statistical analysis

Data were analyzed using PROC mixed procedures of SAS (SAS Inst. Inc., Cary, NC). Kids were divided into two groups according to their breed type (BB and SB). Kid initial and final BW, ADG was analyzed using mixed linear models. Fixed effect investigated included kid breed type. Kid id was modeled as random effect.

*Y_ijk_* = μ + *G_i_* + ε_*ij*_

Where:

*Y_ij_* is the dependent variable

*µ* is the overall mean

*G_i_* is the breed type effect, where *i* = BB or SB

*ε_ij_* is the random error

Carcass traits and meat quality were analyzed using a general linear model of SAS (SAS Inst. Inc., Cary, NC). Kid breed type was treated as the main effect for these traits.

*Y_ij_* = *µ* + *G_i_* + *ε_ij_*

Where:

*Y_ij_* is the dependent variable

*µ* is the overall mean

*G_i_* is the breed type effect, where *i* = BB or SB

*ε_ij_* is the random error

For carcass cuts weights, carcass weight was included as a covariate.

*Y_ij_* = μ + *G_i_* + *bCW* + ε_*ij*_

Where:

*Y_ij_* is the dependent variable

*µ* is the overall mean

*G_i_* is the breed type effect, where *i* = BB or SB

*bCW* linear covariate of carcass weight is the random error

Mean separation for all traits was performed using Tukey’s–Kramer test and decision criterion was determined by the 95% confidence interval for the difference between two means.

## Results and Discussion

### Fattening performance and feed intake

Mean differences for feed intake, birth, initial, and final BW, feed-to-gain ratio, and ADG were small between BB and SB crossbred kids (Table-1). Similarly, no differences in growth parameters between the Anglo-Nubian pure breed and crossbreed Anglo-Nubian. In addition, there were no significant differences between Anglo-Nubian crossbreed, pure Anglo-Nubian, and crossbreed Boer [9]. On the contrary, Offoumon *et al*. showed that the ADG of crossbreds kids presented was better than purebreds [10]. Growth rate of goats is greatly influenced by the sire and dam and mature size, in general, progeny of large breeds grows faster than the progeny of small breeds [11]. Since BB breed has smaller mature body size compared to Shami goat, this could be responsible for reducing the ADG of the crossbred kids. Table-1 shows hot and cold carcass weights and dressing percentage (DP%) traits, results presenting small differences between the two genetic groups. These results are in agreement with Nayga *et al*. [9], the authors stated that both hot and cold carcass weights and DP% did not differ between pure and crossbreed Boer and between pure and cross Anglo-Nubian. However, hot and cold carcass weights and DP% were higher (p<0.01) in Saanen × Hair goat cross than in Alpine × Hair goat and pure Hair goat [12].

**Table-1:** Means, SE, and 95% CI for the difference between the means for growth performance BB and SB kids.

Trait	Breed type^1^		SE	Mean difference	95% CI for the difference between the two means	p-value

	BB	SB				

	Mean	Mean				
Feed intake (kg/d)	1.22	1.17	0.04	0.05	−0.02-0.02	0.46
Birth weight (kg)	3.51	3.65	0.08	−0.14	−0.31-1.2	0.25
Initial weight (kg)	18.89	18.25	0.33	0.64	−0.33-1.61	0.18
Final weight (kg)	29.18	29.97	0.52	−0.78	−2.4-0.8	0.31
ADG (g)	155	156	8.9	−1.0	−0.03-0.03	0.94
Feed-to-gain ratio	8.20	7.86	0.31	0.34	−0.6-1.3	0.44
Hot carcass weight (kg)	13.7	14.1	0.27	−0.38	−1.2-0.44	0.34
Cold carcass weight (kg)	13.21	13.34	0.23	−0.13	−0.8-0.6	0.69
DP%	44.72	45.24	0.90	−0.52	−3.2-2.1	0.68

1 BB=Baladi, SB=Shami-Baladi, CI=Confidence interval, ADG=Average daily gain, SE=Standard error, DP=Dressing percentage

### Carcass traits

Small differences for shoulders, racks, legs, and total offal weights and percentages among the genetic groups were found (Table-2). Gökdal [12] also presented similar results to this study as the genotypes Alpine-Hair and Saanen-Hair males do not have any significant differences in their parts as well. In an another study, no differences were found in shoulders between Boer-Spanish when compared to Spanish-Angora and pure Spanish [13]. On the other hand, there were differences observed between pure Spanish and Spanish-Angora on the leg cut while there were no differences between crossbred Boer when compared to pure Spanish. Conversely, pure Spanish differs significantly when compared to crossbred Spanish-Angora [13]. Similar to the current study, no differences among the carcass offal weight by-products among Honamli, Hair, and Honamli-Hair genotypes [14]. On the contrary, edible slaughter by-products were significantly higher for the Damascus breed than other crossbred kids. The authors related that to higher live BW for Damascus breed compared with other genetic groups [15]. In the present study, absolute mean differences for loin percentage was (0.78) with SB kids having higher values for this trait compared to BB kids. Similar to these results, Anothaisinthawee *et al*. [16] found significant differences in the loin weights between native (Southern Thai), two-way cross (Southern Thai × Anglo-Nubian), and three-way cross (Boer × Anglo-Nubian × Native). On the contrary, there were no differences between pure Honamli and pure Hair when compared by Honamli-Hair kids in relation to their loin cut weight [15]. Since carcass traits are moderate to highly heritable, the advantage of the SB breed type for loin cut percentage could be attributed to breed complementarity.

**Table-2:** Means, SE, and 95% CI for the difference between the means for carcass cut weight and percentage in BB and SB kids.

Trait	Breed type^1^		SE	Mean difference	95% CI for the difference between the two means	p-value

	BB	SB				

	Mean	Mean				
Shoulders weight (kg)	6.12	6.16	0.12	−0.04	−0.37-0.26	0.73
Racks weight (kg)	1.31	1.34	0.02	−0.03	−0.08-0.024	0.27
Loin weight (g)	909	1011	36.0	−102	−0.21-0.005	0.06
Legs weight (kg)	4.4	4.3	0.09	0.1	−0.17-0.34	0.48
Total offal weight (kg)	1.76	1.77	0.065	−0.01	−0.2-0.188	0.95
Shoulders (%)	46.0	46.4	0.82	−0.4	−2.84-2.02	0.72
Racks (%)	9.8	10.1	0.13	−0.3	−0.61-0.16	0.23
Loin (%)	6.9^b^	7.6^a^	0.26	−0.7	−1.5-−0.02	0.045
Legs (%)	33.26	32.55	0.64	0.71	−1.2-2.6	0.44
Total offal (%)	13.3	13.4	0.49	−0.1	−1.55-1.4	0.92

1 BB=Baladi, SB=Shami-Baladi, SE=Standard error, CI=Confidence interval.

a,b Means with different superscripts with the same row differ (p<0.05)

The depth of subcutaneous fat over the *Musculus obliquus externus abdominis* (J), the depth of tissue over the surface of rib at point 110 mm from the midline and area of *M. longissimus* (GR), the depth of subcutaneous fat over the *Musculus latissimus dorsi* at a point at the right angles to the midline (S2), and fat thickness over the ventral edge of *Musculus gluteus medius* (L3) are reported in Table-3. No differences in composition between the two genetic groups were detected. In agreement with this study, no significant differences in fat thickness were found between Boer-Spanish when compared to Spanish-Angora and pure Angora [13]. In addition, this study showed that the *M. longissimus* measurements did not differ in either depth (B) or width (A) in the between the two breed types. However, the *Longissimus* muscle area (cm^2^) between pure Spanish and crossbred Spanish (Spanish × Boer, Spanish × Angora) did not differ (p>0.05). Conversely, there were some differences between pure Angora and Spanish-Angora. This shows that the breed that improved the characteristics of the *Longissimus* muscle area was the Angora breed [13].

**Table-3:** Means, SE, and 95% CI for the difference between the means for fat thickness and *M. longissimus* measurements of BB and SB kids.

Trait	Breed type^1^		SE	Mean difference	95% CI for the difference between the two means	p-value

	BB	SB				

	Mean	Mean				
L3	1.15	1.15	0.093	0	−0. 27-0.27	0.95
S2	1.00	1.05	0.04	−0.05	−0.16-0.06	0.331
J	1.35	1.40	0.13	−0.05	−0.43-0.33	0.785
GR	8.88	8.61	0.27	0.27	−0.53-1.08	0.489
A	49.35	48.95	0.75	0.40	−1.8-2.6	0.709
B	27.8	20.2	6.0	7.6	−10.19-25.5	0.379
C	1.05	1.00	0.04	0.05	−0.06-0.16	0.331

1 L3=Fat thickness over the ventral edge of *Musculus gluteus medius*, S2=The depth of subcutaneous fat over the *Musculus latissius dorsi* at appoint at right angles to the midline, J=The depth of subcutaneous fat over the *Musculus obliquus externus abdominis*, GR=The depth of tissue over the surface of rib at point 110 mm from the midline, A=*Musculus longissimus* width, B *=Musculus longissimus* depth=Baladi, SB=Shami-Baladi, SE=Standard error, CI=Confidence interval. ^a,b^Means with different superscripts with the same row differ (p < 0.05)

Meat quality traits are presented in Table-4. This study concluded that the mean differences for traits were small when comparing between the two breed types; however, high mean differences (2.26) for yellowness (b) were found; SB had higher values than BB kids. In addition, Lopes *et al*. [17] reported that there were no differences between both crossbred and pure kids in SF and CL. On the other hand, crossbred French Alpine-Boer produced higher meat quality (WHC, pH, and color) in comparison with their pure genotypes, respectively [18]. Moreover, significant differences were found in the lightness (a) and redness (a) but no differences in the yellowness (b) between pure Bravia and Serrana and their crosses [19].

**Table-4:** Means, SE, and 95% CI for the difference between the means for meat quality traits of BB and SB kids.

Trait	Breed type^1^		SE	Mean difference	95% CI for the difference between the two means	p-value

	BB	SB				

	Mean	Mean				
CL	38.15	38.21	0.97	−0.055	−2.96-2.84	0.968
WHC	23.89	22.07	0.95	1.82	−1.02-4.7	0.195
SF	9.26	8.82	0.64	0.44	−1.47-2.36	0.632
pH	5.76	5.77	0.018	−0.01	−0.06-0.05	0.816
l	35.78	37.56	1.63	−1.78	−6.66-3.10	0.452
a	2.52	2.25	0.22	0.27	−0.37-0.91	0.387
b	16.74^b^	19.00^a^	0.55	−2.26	−3.86-−0.66	0.008

1 WHC=Water holding capacity, CL=Cooking loss, SF=Shear force, L=Whiteness,

a Redness,

b Yellowness.

2 BB=Baladi, SB=Shami-Baladi, SE=Standard error, CI=Confidence interval, WHC=Water holding capacity

In the present study, as presented in Table-5, none of the results of the leg cut weights indicate differences in crossbred kids. Akbaş and Saatcı [14] showed similar results stating that the long leg weight (g) did not differ among Honamli, Hair, and Honamli-Hair breeds [14]. Carcass and leg compactness index depends on the weight at which the kids were slaughtered rather than on the breed, genotype, or production systems [20]. In agreement with the previous study, there were no differences in the lean percentage in the legs of pure Spanish when compared with crossbred Spanish, while there were significant differences among these three genotypes in the bone and fat percentage of the leg [13].

**Table-5:** Means, SD, and 95% CI for the difference between the means for leg cut of BB and SB kids.

Trait	Breed type^1^		SE	Mean difference	95% CI for the difference between the two means	p-value

	BB	SB				

	Mean	Mean				
Leg weight (kg)	2.19	2.21	0.2	−0.02	−0.071-0.053	0.772
Total muscle (kg)	1.27	1.07	0.103	0.2	−0.108-0.507	0.189
Total bone (g)	564.9	590.1	12.8	−25.2	−63.46-13.04	0.182
Subcutaneous fat (g)	126.6	149.4	14.16	−22.8	−65.1-19.5	0.271
Intramuscular fat (g)	60.9	57.1	5.4	3.8	−12.25-19.87	0.623

1 BB=Baladi, SB=Shami-Baladi, SD=Standard deviation, CI=Confidence interval, SE=Standard error

## Conclusion

Crossing Shami goat with BB did not have a significant value on either growth performance or carcass traits. The only improvement in this mating system was for the percentage and depth of the *Longissimus* muscle. Future studies using the reciprocal cross and under different environmental conditions may reveal different findings.

## Authors’ Contributions

MDO: Designing, supervising, and writing the manuscript. BSO: Helped in statistical analysis and manuscript writing. BN: Helped in technical writing. RA: Drafted the manuscript. All authors read and approved the final manuscript.
